# Health care providers’ adherence to immediate postpartum care guideline and associated factors among women who gave birth in Mekele public teaching hospitals, Tigray 2018

**DOI:** 10.1186/s13104-018-3845-0

**Published:** 2018-10-19

**Authors:** Abera Haftu, Hadgay Hagos, Mhiret-AB Mehari, Brhane G/her

**Affiliations:** Department of Midwifery, Mekele University College of Health Sciences, Tigray, Ethiopia

**Keywords:** Immediate postpartum, Adherence, Mekele

## Abstract

**Objective:**

To assess health care providers adherence to immediate postpartum care and associated factors among women’s who gave birth in Mekele teaching public hospitals, 2018.

**Results:**

The Health care providers’ complete adherence to immediate postpartum care guideline was 22.8%. Health care providers who have complete adherence to prenatal care guideline were 93.3% less likely to have incomplete adherence to immediate postpartum care guideline (AOR 95% CI 0.067 (0.036–0.125)).

## Introduction

According to World health organization (WHO) global estimation of maternal mortality ratio in 2015 was about 216 per 100,000 live births. 99% of the maternal deaths are from developing countries where the sub-Saharan African countries account 66% from the developing countries [1]. Ethiopia is one of the sub-Saharan countries with high maternal mortality which is 412 deaths per 100,000 live births and the nation is failed to achieve Millennium development goal 5 [1].

In order to alleviate the burden of maternal deaths in the globe, world health organization had settled a new plan as part of sustainable development goal (SDGs) where the primary objective is to minimize the maternal mortality ratio to less than 70 per 100,000 live births by the end of 2030 [2], and in the meanwhile at the end of 2030 WHO recommends that no more nation should have MMR of greater than 140 per 100,000 live births [3]. To achieve the goal of SDG, special training should be given to the health care providers who are working in maternal and child health area.

Postpartum follow up has plenty advantages to assess the well being of the mother and neonate [4]. American academy of pediatrics and American college of Obstetrics and Gynecology recommended at least 4–6 visit during the postpartum period irrespective of the women’s age and educational status [5]. The postpartum care follow up mainly focuses on counseling of family planning, initiation of immunization, counseling on breast feeding, detecting of early complication and managing of already occurred maternal and neonatal complications [5]. Even if PPC has enormous benefit still there are many obstacles to access it timely [6]. By 2020 across the globe there is a plan to increase number of women’s to get PPC after they gave birth In which it could enhances the health of the mother and children [7].

Many studies have realized that there a number of challenges to receive postpartum care services properly. Medicaid projects are allowing to get medical services during pregnancy period specially for those with low socio economic status with special needs than the private insurers [8]. Despite of the fact that Medicaid services are important they get failed to cover services that will be provided after delivery like postpartum care which is very essential to the mother and neonate as well [9].

## Main text

### Methods

#### Study are and period

Mekele is the capital city of Tigray region with an area of 24.44 km^2^ and total population of 500,000. It is 783 km far from the capital city of Ethiopia, Addis Ababa. The study population was sampled women’s who gave birth and attended by health care provider in Mekele teaching public hospitals for the period April 1, 2018, to June 30, 2018.

#### Study design

Institution based cross sectional study design was employed.

#### Inclusion and exclusion criteria

*Inclusion criteria* All women’s whose gestational age of greater than or equal to 28 weeks and attended by health care provider in Mekele teaching hospitals were included.

*Exclusion criteria* A woman’s who had arrived in the institution after delivery of the fetus and those who leave from the institution before 6 h of post delivery were excluded.

#### Sample size determination

Sample size was calculated by using single population proportion formula ( *n* = (*za*/2)2(1 − p)/*d*^2^) based on the following assumptions: 95% confidence interval, 5% margin of error and taking 50% magnitude to get maximum sample size on health care providers’ adherence, and 5% for non response rate Hence, the sample size was 403. (1.96)20.5(1 − 0.5)/(0.05)2 = 384 adding 5% for non response rate = 403.

#### Sampling technique

The method of sampling used was simple random sampling from the teaching hospitals (Mekele general hospital and Ayder comprehensive specialized hospital) with 201 and 203 participants from each teaching hospital respectively by 260 and 143 health care providers from Midwives and Nurses consecutively which is one to one assessment of the client to health professionals.

#### Method of data collection

The method of data collection was face to face interview and direct observatory. Face to face interview was used to assess the demographic data of the clients and the observatory technique was used to assess the providers’ adherence to immediate postpartum guide line while they were performing the procedures continuously. To assess the health care providers’ check list was developed which contains fifteen basic elements which was driven from WHO recommendations for the first postpartum follow up. This data collection procedure was carried out by 5° Midwives and it was supervised by two MSC Midwives. The data collector was oriented and trained for 3 days on how to collect the data, which data need to exclude, and about other data collection process. Brief introduction before and during data collection process was given.

#### Data quality control

Data quality was managed by recruiting MSC and BSc holders for each data collection and supervising working in the study site (hospitals) based on experience of data collection and training was given for 3 days on how to collect and supervise the data using the prepared questionnaire. Pretest was done in 40 participants in Wukro Hospital before 3 weeks of actual data collection which was not included in the study and appropriate modification was made to the questionnaire after analyzing the pretest result like time modification, skipping pattern arrangement, wording, and phrases. Daily supervision, spot checking, and reviewing completed questionnaire was conducted to follow for completeness and consistency of the data (questionnaire) by the supervisors and over all data collection process was controlled by principal investigator.

#### Data management and analysis

Data was coded and entered to computer using Epi Data software version 3.1 for its customizing skip benefit and finally data was transferred to SPSS version 20 software package for analysis purpose. Data exploration was undertaken to see if there are odd codes or items that are not logical and then subsequent editing and cleaning were done before analysis. Data was analyzed using SPSS version 20.0 computer software package. Data cleaning and editing were carried out. Frequency with percentage, pie charts, and bar graphs were used to represent results of categorical variables while mean (SD) and median (IQR) were used to represent continuous variables. Binary logistic regression (odds ratio and 95% confidence interval) was used to see the strength of association between dependent variable and each independent variable. Finally, multivariable logistic regression was used to see the predictors of the outcome variable. Variables with p value ≤ 0.25 at binary logistic were further analyzed in the multivariable binary logistic regression. Statistical significance for the association was considered at p value < 0.05. Binary and multiple logistic regressions were done to identify factors associated with term low birth weight neonate. Crude odds ratios were estimated for all independent variables in the binary logistic regression. All independent variables with p value less than 0.25 at bivariate analysis were entered into multivariate logistic regression to control for all possible confounders.

#### Operational definitions

i. Complete adherence: a provider who practice or perform 100% of the proposed checklist.
ii. Immediate postpartum follow up: a follow up given within 6 h of post delivery.

### Results

#### Socio demographic characteristics of the Mothers

The study involved a total of 403 mothers which is 100% in response. The median (± IQR) age of the participants was 27 (± 7 years); 150 (37.2%) of them were in the range of 19–25 years. 362 (89.8%) of the mothers were married and 240 (59.6%) of mothers’ were from urban residence. Of the total participants 330 (81.9%) of them from orthodox religion and almost all 380 (94.3%) were from Tigray region (see Table 1).

**Table 1:** Socio Demographic data of the participants

Variables	Number	Percent
Age		
≤ 18	7	1.7
19–25	150	37.2
26–30	125	31
31–35	75	18.6
≥ 36	46	11.4
Residence		
Urban	240	59.6
Rural	163	40.4
Marital status		
Married	362	89.8
Single	15	3.7
Divorced	24	6
Widowed	2	0.5
Religion		
Orthodox	330	81.9
Muslim	57	14.1
Catholic	12	3
Protestant	4	1
Ethnicity		
Tigray	380	94.3
Amahara	21	5.2
Afar	2	0.5
Monthly income		
< − 500	11	2.7
501–1000	49	12.2
1001–3000	157	39
3001–5000	116	28.8
≥ 5001	70	17.4
Educational level		
No education	73	18.1
Read and write	68	16.9
Primary education	95	23.6
Secondary and above	167	41.4
Spouse educational level		
No education	52	12.9
Read and write	49	12.2
Primary	94	23.3
Secondary and above	208	51.6
Occupation		
House wife	177	43.9
Government employee	80	19.9
Nongovernmental employee	13	3.2
Private organization	120	29.8
Daily laborer	10	2.5
Others	3	0.7
Parity		
Primipara	107	26.6
Multiparas	296	73.4
Gravidity		
Primigravida	71	17.6
Multigravida	332	82.4
Health care providers		
Midwives	260	64.5
Nurses	143	35.5

#### Provider’s adherence to immediate post partum care

In Mekele there are two public teaching hospitals which are Ayder referral hospital and Mekele general hospital, in each of them there were 563 health care providers who are working under MCH areas, among those providers 323 were midwives and 240 were Nurses. Using simple random sampling a total of 403 professionals were recruited 260 &143 from midwives and nurses respectively. Overall the provides adherence to the immediate postpartum check list were 22.8%. Almost all health care providers adhered to recording of the sex of the neonates which is 95%. There is a variation on status of adherence by the health care providers (see Table 2).

**Table 2:** Measuring complete adherence of health care providers to immediate postpartum checklist

Immediate postpartum checklist	Total health care providers N = 403	Midwives N = 260	Nurses N = 143
Sex recording	383 (95%)	247 (95%)	136 (95.1%)
Assessing APGAR score	356 (88.3%)	224 (86.2%)	132 (92.3%)
weight measured	367 (91.1%)	238 (91.5%)	129 (90.2%)
Oxytocin provided	343 (85.1%)	235 (90.4%)	108 (75.5%)
Document mode of delivery	333 (82.6%)	226 (86.9%)	107 (74.8%)
VIT K provided	279 (69.2%)	190 (73.1%)	89 (62.2%)
TTC given	273 (67.7%)	188 (72.3%)	85 (59.4%)
BCG given	195 (48.4%)	111 (42.7%)	84 (58.7%)
Uterine massage done	297 (73.7%)	207 (79.6%)	90 (62.9%)
HC measured	195 (48.4%)	98 (37.7%)	97 (67.8%)
Length measured	199 (49.4%)	102 (39.2%)	97 (67.8%)
Maternal pulse assessed	286 (71%)	178 (68.5%)	108 (75.5%)
Counsel on FP and immunization	338 (83.9%)	224 (86.2%)	114 (79.7%)
Initiate early BF	347 (86.1%)	234 (90%)	113 (79%)
Document providers name properly	311 (77.2%)	193 (74.2%)	118 (82.5%)

#### Determinate factors of health care providers’ adherence to immediate postpartum care guideline

To determine the factors which could be associated with immediate postpartum adherence binary logistic regression was performed and factors which was significant at p-value of ≤ 0.25 was taken to multivariable regression model for controlling of the confounding factors and finally variables with p-value of ≤ 0.05 was considered as significant determinant factors. Age of participants, educational level and income were associated significantly at binary logistic regression but when determine the multivariable analysis, only providers adherence to prenatal was significant factor. Health care providers’ with complete adherence to antenatal care was 93.8% less likely to have incomplete adherence of postpartum care (AOR 0.062 (0.033–0.117)) (see Table 3).

**Table 3:** Multivariable analysis of health care providers’ adherence to immediate postpartum care guide line data from April 1, 2018 to June 30, 2018

Variables	Status of adherence		p-value	COR	AOR	p-value
	Complete	Incomplete				
Adherence to prenatal care						
Complete	44 (10.9%)	291 (72.2%)	0.000	0.063 (0.034–0.116)	0.067 (0.036–0.125)	0.000
Incomplete	48 (11.9%)	20 (5%)	1	1	1	1

### Discussion

In Ethiopia, Midwives and Nurses are the most prioritized professionals to be recruited under maternal and children health caring areas; thus, it is very important incapacitating of them both in knowledge and skill at providing of postpartum care to reduce maternal and neonatal mortality in remarkable way.

In this study the overall health care providers’ adherence to immediate postpartum care guideline was about 22.8%. The adherence of the health care providers towards to sex of the neonate was 95% and among the professionals the adherence was almost similar which was 95% and 95.1% in midwives and nurses respectively. Total adherence of the health care providers towards to weight measurement, Oxytocin administration, VITK and TTC was 91.1%, 85.1%, 69.2% and 67.7% respectively. Among the above mentioned parameters midwives adherence was a bit higher than the nurses. Overall the providers’ adherence to fetal length and head circumference measurement was below 50% which is 49.4% and 48.4% respectively.

In the multivariable analyses of this study provider with incomplete adherence to antenatal care guideline was less likely to adhere to the postnatal care guideline and this is consistent with other studies which showed that prenatal care guideline was the strongest predictor of postpartum care utilization [10–12]. This similarity could be because of the tool we use is standardized which is driven from WHO and SMP guidelines. Unlike to other studies low income, marital status and unplanned pregnancy was not determinant factors for provides’ adherence to immediate postpartum guideline [11, 12].

In this study the overall adherence of the providers is low based on the fifteen standardized checklist but when come to each items adherence in some of them they performs in good manner. The providers’ assessing to neonates weight, giving Oxytocin, TTC and BCG was 91.1%, 85.1%, 69.2%, 67.7% and 48.4% respectively. More than half of them failed to assess the neonates’ length (50.6%) and the providers’ status of documenting the provider name properly was about 22.8%.

Antenatal period is a good time to educate and counsel the pregnant woman about the advantages of postpartum care. If a woman ones fulfill a criteria to attend postpartum follow up, the services that would be covered during the follow up are assessing of uterine involution, maternal condition, fetal condition and contraceptive counseling [13]. Maternal and new born care are taken as one of the ten essential health benefits in well standardized and established health care services [14].

## Limitation

- The study environment was teaching hospitals.
- Many students who are not yet graduated and failed to adhere to the guideline.
- Negligence of the health professionals because of population density.
